# American Origin of Syphilis

**Published:** 1844-04

**Authors:** 


*American Origin of Syphilis.*—Although the question, whether syphilis existed in the West Indies and on the continent of America at the period of their discovery by the Spaniards, is, by the profession in this country, very generally decided in the negative; yet, in order effectually to remove all remaining doubts, and to efface completely the stigma thus cast upon the New World by the Old, at a time when, in Europe, the opinion that the human species, and indeed the whole animal kingdom, in America, had become entirely *degenerated*, we will here present the evidence of two of the most accomplished and distinguished historians of modern times. Peculiarly qualified for this decision by the nature of their literary pursuits, it will suffice to say that the one is the author of "The Life and Voyages of Columbus," and the other, of "Ferdinand and Isabella," and more recently, "The History of the Conquest of Mexico." After this testimony shall have been presented to the world, this long-mooted question must be considered as conclusively put to rest, and never more to be revived.

The following letter is addressed by Mr. Prescott to Alexander E. Hosack, M.D.

"*Boston, January* 22d, 1844.

"*My Dear Sir:* I have received your note of the last week, inquiring whether, in my researches relative to the History of Mexico, I had met with any trace of the venereal disease among the Aborigines previous to the coming of the Spaniards. I observe, also that you remark that Dr. Forry had already put the same question in a letter addressed to me some time since. I assure you that I never received that letter.

If I had, I should have answered it with the sincere pleasure and promptness with which I now reply to yours.

"In a note in the 'History of Ferdinand and Isabella,' I took occasion to express my own conviction that the venereal disease did not exist among the natives of America at the time of its discovery. I had met with no allusion to it in the narratives of Columbus or his son Ferdinand, or in any other record of the Spanish adventures.

"I have been led into a much wider range of observation in preparing the 'History of the Conquest of Mexico.' But it has served to confirm my former opinion, since I have never met with a notice of this disease, or of any which resembles it. The ancient chronicles speak of an Indian epidemic, called the *Matlazahuatl*, which swept off great numbers of the natives both before and after the Conquest, and which seems to have had some resemblance to the yellow fever. They also notice the introduction of the small-pox by a black, who came into the country the year after the arrival of Cortés. The Spaniards would certainly not have omitted to notice so terrible a disorder as the venereal, had it been found among the natives; especially, as considering their own licentious indulgence, it must have fallen very heavily on themselves. Their uniform silence, therefore, is evidence so strong, that it may be called positive, rather than negative, and may be considered as establishing the fact that the disease was not known in the Mexican empire at the time of its discovery. Whether a disease so easily propagated among adjacent tribes, and which seems to be circumscribed by no parallel of latitude, could have existed in other parts of the Continent without finding its way into Mexico, is a question which your own knowledge of the subject will enable you to determine better than I can.

"Believe me, my dear sir, very truly yours,

"WM. H. PRESCOTT.

"Alex. E. Hosack."

We here present the note of Mr. Irving, written just on the point of his departure as Minister to Spain:

"*New York, March* 22, 1842.

"*Dear Sir:* In the hurry of various occupations preparatory to my departure, I find that I have your note, of March 12th, lying unanswered. I have not now time to refer to papers and documents to refresh my memory on the subject of your inquiry. I recollect, however, that my impression was, while writing the History of Columbus, that the disease you mention did not exist among the natives of America at the time of the discovery, nor among the voyagers during the first voyages; but that it originated in Europe. When I have leisure, I will look into the matter in Madrid; and should I discover any facts upon the subject, I will communicate them to you.

"Very respectfully, &c.,

"WASHINGTON IRVING.

"Dr. Jno. Watson."

[*New York Med. Jour.*

